# Solar Hydrogen Production from Cost Effective Stannic Oxide Under Visible Light Irradiation

**DOI:** 10.1186/s11671-019-3127-3

**Published:** 2019-08-30

**Authors:** Yingnan Duan, Wanliang Yang, Wei Zheng, Guiwei He, Meng Chen, Mengkui Tian

**Affiliations:** School of Chemistry and Chemical Engineering, Guizhou University, Guiyang, Guizhou 550025 People’s Republic of China

**Keywords:** Photocatalyst, Water splitting, Stannic oxide, Photoelectrochemical

## Abstract

Visible-light-driven stannic oxide was synthesized by facile one-pot solvothermal method from SnCl_2_·2H_2_O and methanol. The as-prepared powder was identified by XRD as the low crystalline phase of SnO_2_, and its absorption edge reached about 530 nm, presenting good potential to respond to visible light. Under visible light irradiation (λ > 420 nm), the as-prepared tin oxide showed good anodic photocurrent effects on FTO photoelectrode, and showed hydrogen and oxygen evolution activities under electron donor (methanol) and acceptor (AgNO_3_), respectively, even without any co-catalyst loading. The visible-light-driven mechanism for this SnO_2-x_ maybe ascribed to Sn^2+^ self-doped into Sn^4+^ and formed an energy gap between the band gap of SnO_2_.

## Introduction

Acquisition of clean hydrogen energy by splitting of water using plentiful solar energy is considered as an ideal way to resolve the global renewable energy demand and environment problems [1–4]. In particular, photocatalytic or photoelectrochemical splitting water is one of the most ideal ways considering resource sustainability, environmental, and cost issues [5, 6]. The urgent work for water splitting by photocatalysis is to design and develop semiconductor photocatalysts with appropriate band gap to make best use of solar energy and band edges to meet oxidization and reduction water requirement as well as high quantum yield and high stability [7]. Up to now, the development of photocatalysts experienced from binary oxides (TiO_2_, ZnO, Fe_2_O_3_) [8], ternary oxides (SrTiO_3_, K_4_Nb_6_O_17_, NaTaO_3_) [9], to multi elements compounds (K_4_Ce_2_M_10_O_30_(M = Ta, Nb) [10], especially solid solution compounds (GaN: ZnO, ZnGeN_2_-ZnO) [11], and series of (oxy) nitrides (Ta_3_N_5_, TaON, LaTiO_2_N) [12, 13], (oxy) sulfides (Sm_2_Ti_2_S_2_O_5_, Cu_2_ZnSnS_4_) [14] based on band engineering methods, as well as from p block photovoltaic cell semiconductors candidates such as GaInP/GaAs, GaPN, GaAsPN, p-InGaN, etc. [15]. Additionally, the morphologies of film or powder with nanowire, nanorod/nanotube, and nanobelt etc. are extensively controlled [16]. Unfortunately, most of them failed to satisfy the mentioned above requirements simultaneously.

SnO_2_ is a well-known semiconductor with band gap about 3.6–3.8 eV. However, wide band gap and low conduction band edge (more positive than that of H^+^/H_2_) of SnO_2_ restrict its utilization as a photocatalyst for water splitting [17]. In the most cases, SnO_2_ was used as a part of composite or coupled photocatalysts, such as in SnO_2_-TiO_2_ [18], SnO_2_-ZnO [19] for its lower conduction band edges to facilitate the transferring photo-generated electrons from the host photocatalyst.

In this communication, visible-light-driven SnO_2-x_ was synthesized by facile one-pot solvothermal method from the precursors of SnCl_2_·2H_2_O. The as-prepared powder was identified through X-ray diffraction (XRD) as pure SnO_2_ phase, and ultraviolet–visible spectroscopy (UV-vis) spectrum indicated its absorption edge about 570 nm, corresponding to band gap of 2.17 eV, presenting good potential to respond to visible light. The photoelectrochemical and photocatalytic water splitting activities under visible light were presented.

## Methods

### Reagents

All chemicals of analytical grade were purchased from Sinopharm Chemical Reagent Co., Ltd., Shanghai, China, and used as received without further purification. The super pure water (18.25 MΩ cm) was used as solvent for photoelectrode preparation and photocatalytic measurement.

### Preparation of Powder SnO_2-x_

SnO_2-x_ was prepared by conventional solvothermal method with 0.02 mol SnCl_2_·2H_2_O (SnCl_4_·5H_2_O) dissolved into 100 mL methanol solvent and stirring for 30 min. Then, adjusting the pH value from initial 1.0 to 3.0 by dipping 0.02 mol/L NH_3_·H_2_O slowly with stirring, getting white floccule. After reacting for 2 h, the mixture was transferred into a 200 mL Teflon-lined autoclave and heated at 423 K for 20 h. The yellow slurry was obtained from washing with deionized water and ethanol several times, and dried at 343 K for 12 h, got the targeted sample.

### Preparation of SnO_2-x_ Electrode

Porous thin film electrodes were prepared by electrophoretic deposition method on conductive fluorine-doped tin oxide glass (FTO, Ahahi Glass Co.). The electrophoretic deposition was carried out in an acetone solution (40 mL) containing as-prepared powder (40 mg) and iodine (15 mg), which was dispersed by sonication for 3 min. The coated area was controlled to be ca. 1.5 × 4 cm. This procedure resulted in the formation of SnO_2-x_ layer with uniform thickness of ca. 2 μm, with good reproducibility.

### Photocatalytic Evaluation

The photoelectrochemical measurement was performed by three-electrodes configuration mode consisted of a working electrode (prepared electrode), a counter electrode (Pt mesh), and a reference electrode (Ag/AgCl) as well as electrolyte (0.1 M aqueous Na_2_SO_4_ solution) on electrochemical workstation (Autolab PGSTAT 204, Switzerland), and the pH value of the electrolyte solution was adjusted to 4.05 by 0.1 M H_2_SO_4_. The solution was purged with Ar for over 10 min before the measurements. The electrodes were irradiated through silicon glass window by a Xe lamp (300 W, Cermax) fitted with a cut-off filter (Hoya L-42) to block light of wave length less than 420 nm.

The photocatalytic activities were carried out in a Pyrex side-irradiation-type reaction vessel connected to a glass closed gas circulation system. A flow of cooling water was used to maintain the reaction system at room temperatures. Then, 0.2 g powder was dispersed into 200 mL solution, irradiated by 300 W Xe-lamp fitted with a cut-off filter (Hoya L-42) to block light of wave length less than 420 nm. The evolved gas was analyzed by gas chromatography with thermal conductivity detector (TCD) detector and Ar as carrier.

### Characterizations

The sample was identified by X-ray powder diffraction on Geiger-flex RAD-B, Rigaku; Cu Kα). Scanning electron microscopy (SEM) images were obtained on field-emission scanning electron microscopy (FE-SEM; S-4700, Hitachi). UV-vis diffuse reflectance spectrum was recorded by spectrophotometer (JASCO, V-670). The Brunauer-Emmett-Teller (BET) surface area was measured using a BELSORP-mini instrument (BEL Japan) at 77 K. The elements and valence states of the samples were analyzed by X-ray photoelectron spectroscopy (XPS) (Thermo Fisher K-Alpha, America). Transmission electron microscope (TEM) and high-resolution transmission electron microscopy (HRTEM) images of samples were performed on Tecnai G2 F20 transmission electron microscopy at 200 kV accelerating voltage.

## Results and Discussion

**Fig. 1:**
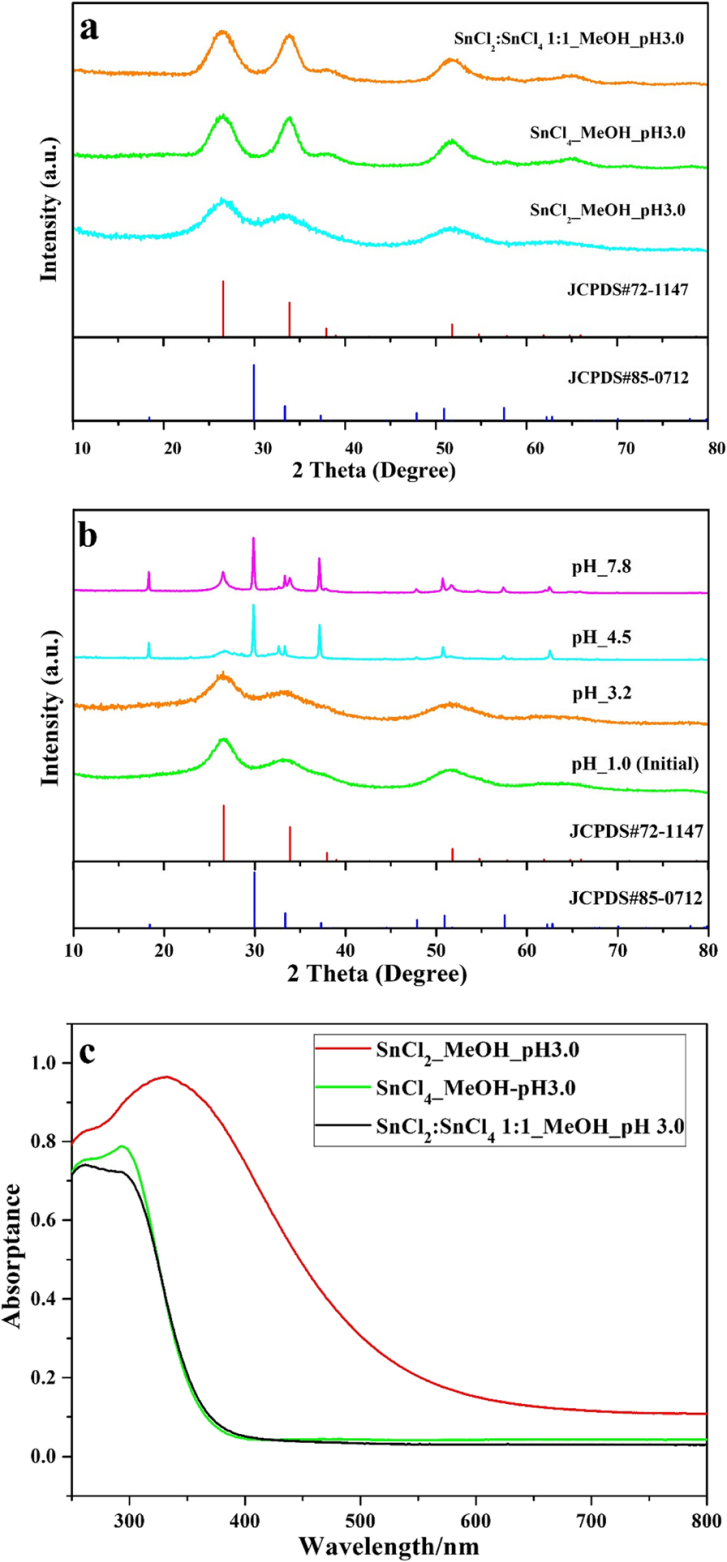
XRD patterns of prepared SnO_2_ **a** with different precursors, **b** different pH values of SnCl_2_·2H_2_O as precursor (JCPDS#72-1147 and 85-0712 of SnO_2_ and SnO), and **c** UV-vis DRS spectra with different precursors

**Fig. 2:**
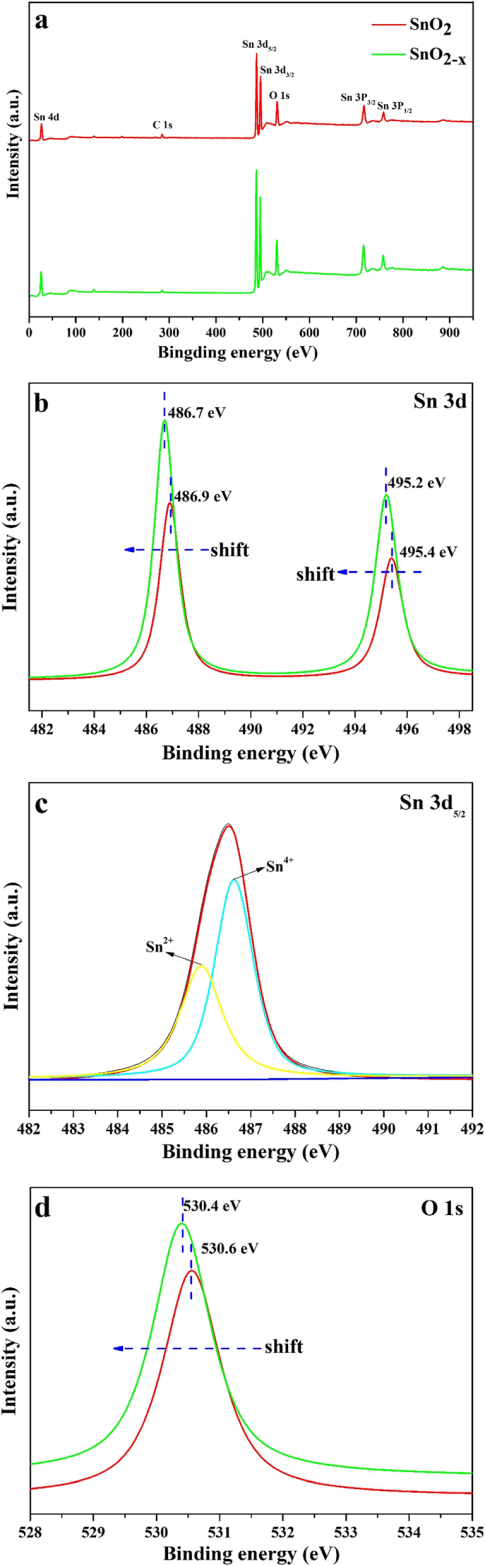
Survey XPS spectra (**a**), the Sn 3d XPS spectra (**b**), the O 1s XPS spectra (**d**) of SnO_2_ and prepared SnO_2-x_, and **c** the Sn 3d_5/2_ XPS spectra of prepared SnO_2-x_

The as-prepared powder was identified by XRD patterns. The compositions, absorption properties, and crystallite of as-prepared samples closely depended on the preparation conditions, such as tin precursors (SnCl_2_·2H_2_O, SnCl_4_·5H_2_O), pH values, and consequently further heat treatment. As an example, this sample prepared by SnCl_2_·2H_2_O with methanol as solvent and adjusted pH value to 3.0 by NH_3_·H_2_O, XRD pattern identified its pure SnO_2_ phase with poor crystalline (Fig. 1a), and UV-vis spectrum (Fig. 1c) revealed its absorption edge is about 570 nm, corresponding to band gap of 2.17 eV, showing great potential to response to visible light. While for these SnO_2_ from precursors of SnCl_4_·5H_2_O and SnCl_4_·5H_2_O with SnCl_2_·2H_2_O (molar ration 1:1) under the same procedures above, their absorption edges are almost the same at about 370 nm. Moreover, with the precursor SnCl_4_·5H_2_O, we cannot get visible-light-driven SnO_2_ by co-precipitation method in air and by hydrothermal method in water. Furthermore, for precursor SnCl_2_·2H_2_O in methanol solvent, with the increase of pH value, the obtained powder became the mixture of SnO_2_ and SnO (Fig. 1b). The XPS of the as-prepared powder was measured to characterize the elemental compositions and chemical states, as shown in Fig. 2. The survey scan spectra (Fig. 2a) of the SnO_2_ and SnO_2-x_ (SnCl_2_·5H_2_O as precursor) sample clearly indicate the obvious peaks of Sn, C, and O. Figure 2b showed that the binding energy of Sn 3d in SnO_2-x_ decreased by 0.2 eV as compared to pure SnO_2_ (from 486.9 to 486.7 eV for Sn 3d_5/2_, and from 495.4 to 495.2 eV for Sn 3d_3/2_). As shown in Fig. 2c, the Sn 3d_5/2_ signal of SnO_2-x_ sample centered at 486.7 eV can be deconvoluted by the multi-Gaussian function into two parts centered at 486.8 and 485.8 eV assigned to Sn^4+^ and Sn^2+^, which confirmed the presence of Sn^2+^ dopants in the prepared SnO_2-x_ because of the formation of oxygen vacancies which (cut down) the binding energy of Sn 3d to preserve charge neutrality [20]. Figure 2d showed that O 1s transition peak shifted 0.2 eV (from 530.6 to 530.4 eV) upon self-doping of Sn^2+^, and the formation of oxygen vacancies was also considered to enhance the absorption in the visible-light region [21, 22]. The optical absorption properties of prepared SnO_2-x_ as different precursors were studied by UV-vis DRS spectroscopy (Fig. 1c). The visible light response ability of prepared SnO_2-x_ by SnCl_2_·2H_2_O as precursor was attributed to the incorporation of Sn^2+^ into the lattice of SnO_2-x_ [20]. These obvious differences in control preparation conditions indicated that the visible-light-driven mechanism for as-prepared SnO_2-x_ had great relation with Sn^2+^ species in oxygen inefficient situation.

**Fig. 3:**
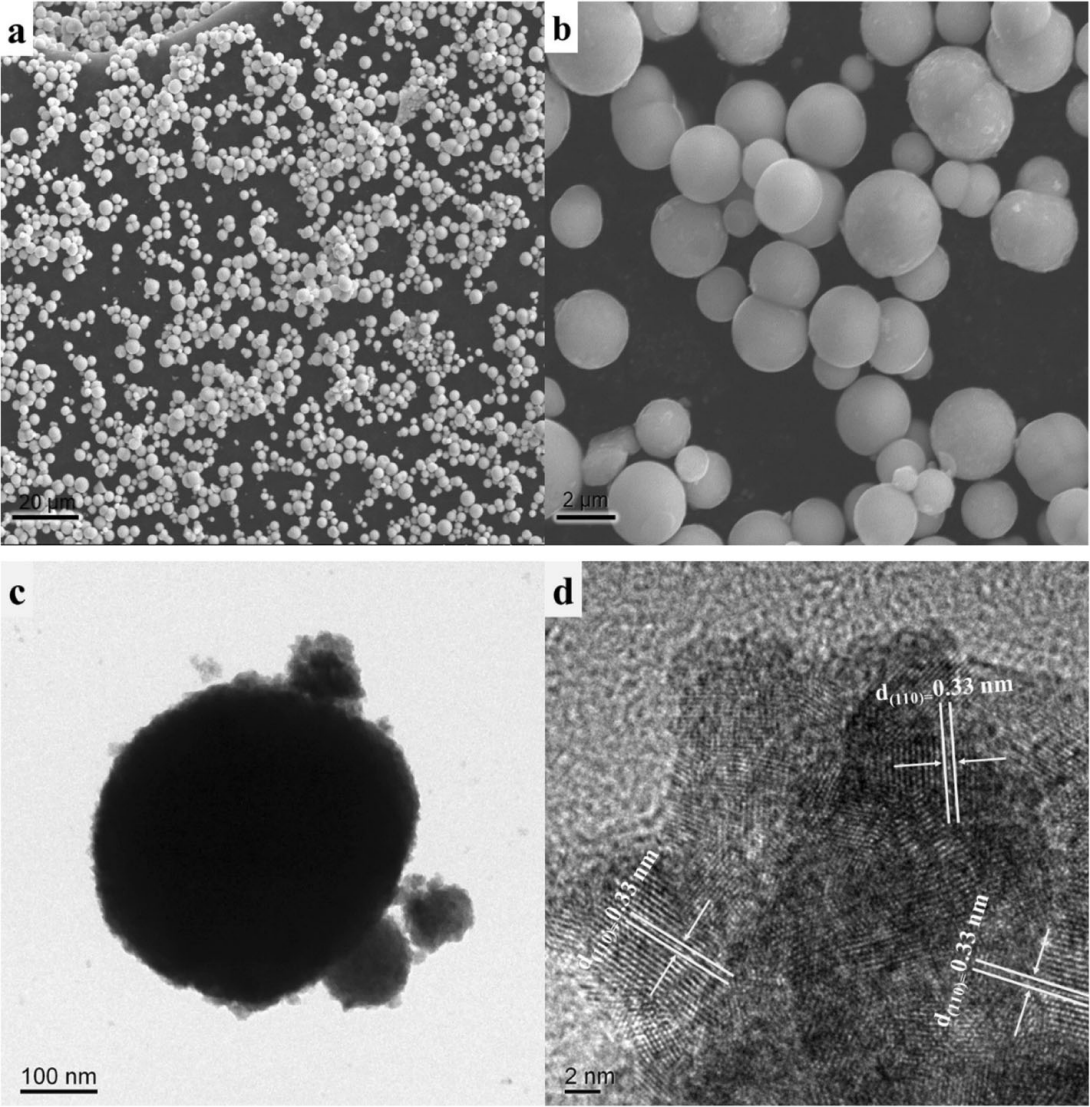
SEM (**a** and **b**), TEM (**c**), and HR-TEM (**d**) images of prepared SnO_2-x_

The microstructure of prepared SnO_2-x_ was obtained by SEM, TEM, and HRTEM. The SEM images illustrated regular spherical particle in diameter of about 1–2 μm (Fig. 3a, b), while their BET surface areas are about 100 m^2^/g, and the crystal size is about 2.5 nm from BET measurement, which is consistent with that from calculation by Scherrer equation. As shown in Fig. 3c, we can see that prepared SnO_2-x_ showed regular spherical particle consisted with SEM image. The HRTEM image (Fig. 3d) indicated that the lattice fringes measured with a spacing of 0.33 nm were clearly visible, corresponding to the (110) atomic plane of SnO_2_ with a tetragonal cassiterite phase.

**Fig. 4:**
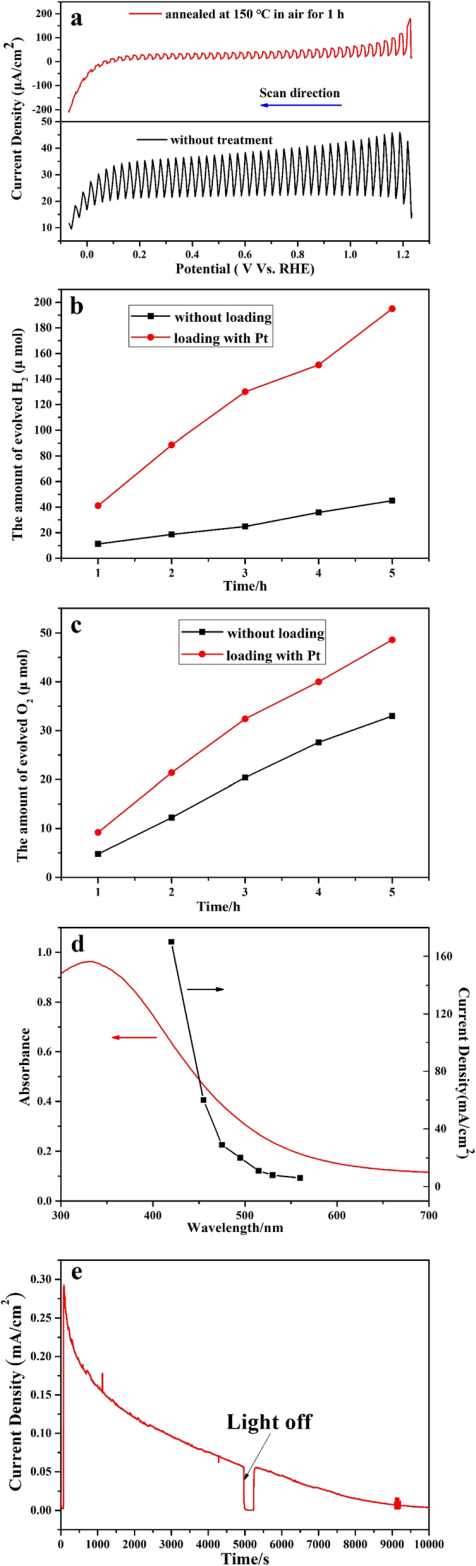
Photocurrent effect of prepared SnO_2-x_. **a** The photocatalytical activities of H_2_ evolution (**b**) and O_2_ evolution (**c**). The wavelength dependence on photocurrent effect for prepared SnO_2-x_ (**d**). **e** The I-T curve for this parpared SnO_2-x_

The photocurrent effect on as-prepared SnO_2-x_ electrode under visible light (λ > 420 nm) was shown in Fig. 4a. For this photoelectrode without any treatment, although there showed obvious photoanodic current, a N-type semiconductor responsive characters, the photocurrent properties are not so normal in slow increase and decrease responsive to light on and off, which may be ascribed to the surface capacity effect. For this with further heat treatment at 150 °C in air, there showed not only the increase of current density, but also the improvement of its photocurrent properties. From Fig. 4a, the as-prepared SnO_2-x_ posed with onset potential less than 0 V Vs reversible hydrogen electrode (RHE), that is to say, the as-prepared SnO_2-x_ with conduction band located negative than that of H^+^/H_2_, indicating that the as-prepared SnO_2-x_ can split water without bias potential. To make certain the potential of the band edges for as-prepared SnO_2-x_, the photocatalytic water decomposition in powder for half reaction under visible light was carried out in a gas circular system. As shown in Fig. 4b, c, the as-prepared SnO_2-x_ demonstrated obvious H_2_ and O_2_ evolution activities under visible light irradiation(λ > 420 nm) with the presence of electron donor (methanol) and acceptor (AgNO_3_) respectively even without any co-catalyst loading and modification. And with the loading of Pt (1 wt.%) by in-situ photo-deposition method from H_2_PtCl_6_, the activities were prompted greatly. The hydrogen and oxygen evolution activities under visible light further confirmed that the as-prepared SnO_2-x_ poses appropriate band edges to meet the requirement for water redox reaction. The wavelength dependence on photocurrent (Fig. 4d) showed good agreement with absorption edge, indicating the band transition properties. The photocurrent density of prepared SnO_2-x_ time dependance was measured under visible-light irradiation at a bias potential of 0.6 V Vs RHE (Fig. 4e). After 10,000 s of irradiation, the photocurrent density is slowly reduced to zero. It can be found that the stability of prepared SnO_2-x_ is poor which is due to the oxidation of Sn^2+^.

**Scheme 1:**
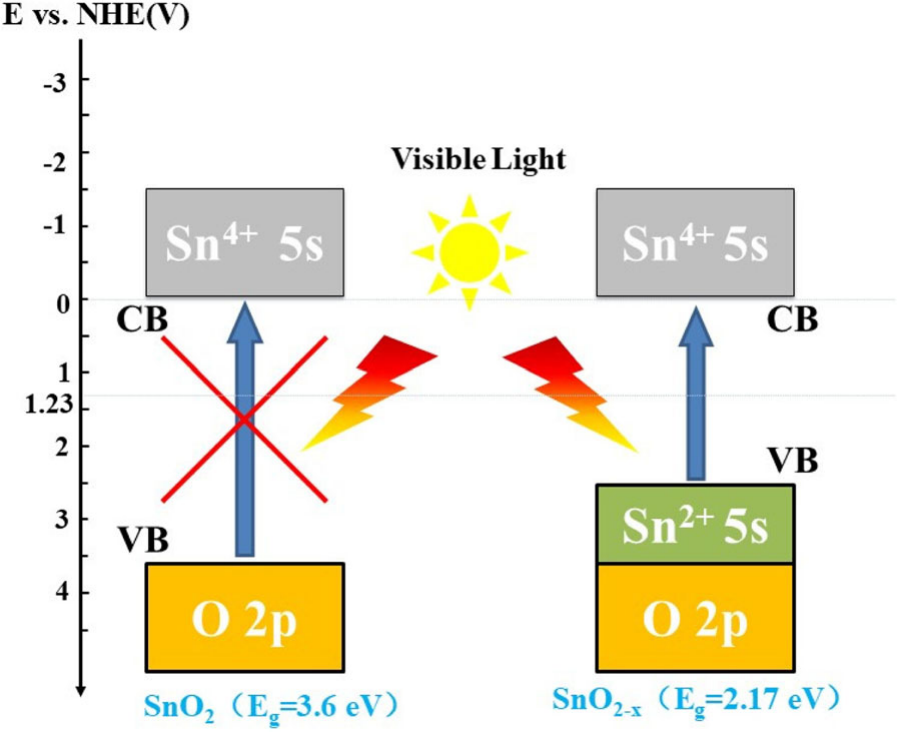
Schematic diagram for the band structure of pure SnO_2_ and prepared SnO_2-x_ photocatalyst

SnO_2_, a known wide band-gap semiconductor, phases with different oxygen composition. Non-stoichiometry of SnO_2_, in particular oxygen deficiency or impurity dopants, can donate electrons into the conduction band, and the conduction band is a single band of s-type character that is strongly dispersed with a minimum at the T-point of the Brillouin zone, which make it a good electron conduction [23]. Additionally, for these visible-light-driven Sn^2+^ including compounds Sn_2_Nb_2_O_7_ (SnNb_2_O_6_), and Sn^2+^ ion-exchange Sn^2+^/K_4_Nb_6_O_17_, Sn^2+^/KTiNbO_5_, it was ascribed that the Sn 5 s^2^ contributes to the top of the valence band, and locates in about 0.7~1.4 eV negative than that of O 2 p [24]. So here, for as-prepared SnO_2-x_, the visible-light-driven mechanism maybe ascribed to the energy levels that are formed between Sn^2+^ 5 s orbital and O 2p orbital. On the other hand, the valence state of Sn^2+^ is more negative than that of Sn^4+^ (illustrated in Scheme 1) resulting in doping in the lattice that will cause charge imbalance to form oxygen vacancies, which has an effect on the surface properties and charge transfer of the catalyst.

## Conclusion

Cost-effective stannic oxide photocatalyst has been successfully synthesized by facile one-pot solvothermal method from SnCl_2_·2H_2_O and methanol. It is significant to show visible light responsive ability and photoelectrolysis water decomposition activities. The visible-light-driven mechanism for this SnO_2-x_ maybe ascribed to self-doping by Sn^2+^ generating oxygen vacancies to preserve charge neutrality which can enhance the performance of photocatalyst. Further work focusing on the improvement of activities and stability are under investigations.

## Data Availability

All data generated or analyzed during this study are included in this published article.

## Abbreviations

BET: The Brunauer-Emmett-Teller
CB: The conduction band
E_(RHE)_: E (Ag/AgCl) + 0.0591pH + 0.197)
FTO: Fluorine-doped tin oxide glass
HRTEM: High-resolution transmission electron microscopy
RHE: Reversible hydrogen electrode
SEM: Scanning electron microscopy
TCD: Thermal conductivity detector
TEM: Transmission electron microscope
UV-vis: Ultraviolet–visible spectroscopy
XPS: X-ray photoelectron spectroscopy
XRD: X-ray diffraction
